# The Novel Small Molecule BTB Inhibits Pro-Fibrotic Fibroblast Behavior though Inhibition of RhoA Activity

**DOI:** 10.3390/ijms231911946

**Published:** 2022-10-08

**Authors:** Ashley R. Rackow, David J. Nagel, Gregory Zapas, Ryan S. Clough, Patricia J. Sime, R. Matthew Kottmann

**Affiliations:** 1 Division of Pulmonary Disease and Critical Care Medicine, University of Rochester Medical Center Rochester, Rochester, NY 14642, USA; 2 Department of Human Genetics, University of Utah Salt Lake City, Salt Lake City, UT 84112, USA; 3 Division of Pulmonary Disease and Critical Care Medicine, Virginia Commonwealth University Richmond, Richmond, VA 23284, USA

**Keywords:** pulmonary fibrosis, lung, GPR65, fibroblast, GPCR, non-canonical TGFβ signaling

## Abstract

Idiopathic pulmonary fibrosis (IPF) is a progressive, chronic, interstitial lung disease with a poor prognosis. Although specific anti-fibrotic medications are now available, the median survival time following diagnosis remains very low, and new therapies are urgently needed. To uncover novel therapeutic targets, we examined how biochemical properties of the fibrotic lung are different from the healthy lung. Previous work identified lactate as a metabolite that is upregulated in IPF lung tissue. Importantly, inhibition of the enzyme responsible for lactate production prevents fibrosis in vivo. Further studies revealed that fibrotic lesions of the lung experience a significant decline in tissue pH, likely due to the overproduction of lactate. It is not entirely clear how cells in the lung respond to changes in extracellular pH, but a family of proton sensing G-protein coupled receptors has been shown to be activated by reductions in extracellular pH. This work examines the expression profiles of proton sensing GPCRs in non-fibrotic and IPF-derived primary human lung fibroblasts. We identify TDAG8 as a proton sensing GPCR that is upregulated in IPF fibroblasts and that knockdown of TDAG8 dampens myofibroblast differentiation. To our surprise, BTB, a proposed positive allosteric modulator of TDAG8, inhibits myofibroblast differentiation. Our data suggest that BTB does not require TDAG8 to inhibit myofibroblast differentiation, but rather inhibits myofibroblast differentiation through suppression of RhoA mediated signaling. Our work highlights the therapeutic potential of BTB as an anti-fibrotic treatment and expands upon the importance of RhoA-mediated signaling pathways in the context of myofibroblast differentiation. Furthermore, this works also suggests that TDAG8 inhibition may have therapeutic relevance in the treatment of IPF.

## 1. Introduction

Idiopathic pulmonary fibrosis (IPF) is a progressive interstitial lung disease affecting approximately 3 million people worldwide [1]. Although the pathogenesis of IPF is not entirely established, the current paradigm postulates that repeated epithelial injury leads to sustained and aberrant wound healing, which ultimately results in the deposition of excess scar tissue in the interstitial space [2]. One key cellular effector of fibrosis is the fibroblast. Fibroblasts are primarily responsible for the production of excess matrix proteins in IPF [3,4,5,6]. In response to injury, fibroblasts differentiate into myofibroblasts, becoming contractile and expressing alpha smooth muscle actin (αSMA) [7].

Myofibroblasts exhibit altered pro-fibrotic behaviors and experience metabolic reprogramming which accompany differentiation [8,9,10]. Myofibroblasts increase glycolytic enzyme expression, which results in increased production of lactate [8]. Our prior work demonstrated that the endogenous production of lactate by fibroblasts serves to perpetuate myofibroblast differentiation. Critically, inhibition of lactate dehydrogenase A (LDHA), the enzyme responsible for the conversion of pyruvate to lactate, prevents myofibroblast differentiation in vitro [11,12] and fibrosis in vivo [13,14]. Thus, the upregulation of LDHA in myofibroblasts leads to increased lactate production and export, which lowers extracellular pH and is likely responsible for the decreased pH of fibrotic lung tissue in vivo [15].

Although lactate induces myofibroblast differentiation, this effect is abolished when media containing lactate is pH adjusted to neutral [8]. Our prior work suggested that lactate induces myofibroblast differentiation via pH mediated activation of the pro-fibrotic cytokine transforming growth factor beta (TGFβ). TGFβ is held in a latent complex in the extracellular space, requiring activation in order to be released and bind its receptor to exert its pro-fibrotic effects [16,17,18,19]. Decreases in extracellular pH represent one mechanism by which TGFβ can become activated [20]. We therefore hypothesize that cellular mechanisms which promote extracellular acidification will be pro-fibrotic while those that suppress extracellular acidification will prevent myofibroblast differentiation and fibrosis.

In the early 2000s, a group of proton sensing GPCRs was discovered [21,22,23]. Although there is variation amongst family members, it is proposed that these receptors sense physiologic pH through histidine residues on their extracellular domains [23]. The family includes T-cell death-associated gene 8 (TDAG8 or GPR65), ovarian cancer G-protein coupled receptor 1 (OGR1 or GPR68), G2 accumulating protein (G2A or GPR132), and GPR4. None of these receptors have been examined in the context of fibrosis and there is very little data in fibroblasts or primary human cell types. Here, we examine the expression profiles of the proton sensing GPCRs in primary human lung fibroblasts derived from patients with IPF and non-fibrotic donors. Although the precise roles of the proton sensing GPCRs are not known, we hypothesized that pH sensing GPCRs that are upregulated in IPF fibroblasts and lung tissue would be pro-fibrotic. We identified TDAG8 as the only proton sensing GPCR that is upregulated in IPF fibroblasts. TDAG knockdown was performed, revealing that a loss of TDAG8 in fibroblasts inhibits myofibroblast differentiation. To better understand the role of TDAG8 in fibroblast biology, we sought to identify modulators of the receptor. The only available small molecule known to directly interact with TDAG8 was BTB09089 (BTB). BTB is a small nonpolar organic molecule with three conjugated rings and was originally identified as a positive modifier of TDAG8 activity, though initially its interaction with the receptor was not described [24]. Further molecular docking studies revealed that BTB binds allosterically to TDAG8, and three residues specifically (Arg187, Phe242, and Tyr272) were required for this interaction [25].

To illuminate the role of TDAG8 activation in fibroblasts, we utilized BTB. As BTB was described as a positive allosteric modulator (PAM) for TDAG8, we anticipated that BTB would activate TDAG8 and thereby induce myofibroblast differentiation. Contrary to our hypothesis, we discovered that BTB potently inhibits myofibroblast differentiation, matrix production and fibroblast proliferation. Subsequently, we identified that BTB did not inhibit myofibroblast differentiation via activation of TDAG8. Thus, although BTB may be a PAM of TDAG8, the off-target effects of BTB predominate. Our data suggest that the anti-fibrotic effects of BTB are related the inhibition of RhoA mediated signaling pathways, including the downstream mediators ROCK1 and mDia.

This work highlights the novel small molecule BTB, as an inhibitor of myofibroblast differentiation. Furthermore, this work suggests that the development of TDAG8 inhibitors may have significant therapeutic potential. This manuscript also highlights the importance of diverse RhoA signaling in myofibroblast differentiation. Overall, the work builds towards the development of pH targeted therapeutics which may prove efficacious in the treatment of IPF and other progressive fibrotic diseases.

## 2. Materials and Methods

Cell Culture and Reagents: Primary human lung fibroblasts were isolated from biopsy as previously described [26]. The diagnosis of IPF was made on the basis of the ATS consensus statement including the identification of definite or probably UIP on biopsy [27]. All donors gave written, informed consent. Cells were cultured with 10% FBS (Sigma-Aldrich, Darmstadt, Germany), 1% L-glutamine, and 1% antibiotic-antimycotic in Eagles Minimum Essential Media (Gibco, New York, NY, USA). TGFβ was purchased from R&D Systems and was used at a dose of 1 ng/mL. BTB09089 was ordered from MolPort (002-892-881, 10–50 µM). Non-targeting control siRNA and TDAG8 siRNA were purchased from Origene and utilized at a final dose of 50 nM per well. Transfections were performed with X-treme Gene siRNA transfection reagent (Millipore-Sigma, Darmstadt, Germany) in basal media without added antibiotics.

Transfections: For siRNA transfections, fibroblasts were seeded at 70% confluency and given 24 h to adhere. Subsequently, fibroblasts were transfected with 50 nM non-targeting siRNA to siRNA specific to TDAG8 (Origene, Rockville, MD, USA). The transfection reagent and siRNA constructs were incubated for 20 min in basal media before the mixture was applied to cells. The media was replaced 18 h after transfection, and protein was harvested 96 h after transfection.

Western blotting and Slot blotting: Cell lysates were run on an SDS-PAGE gel and transferred using the Trans-Blot Turbo Transfer System (Bio-Rad, Hercules, CA, USA). Western blots were blocked using EveryBlot Blocking Buffer (Bio-Rad) and imaged using the Chemidoc MP fluorescent imager (Bio-Rad). Western blots were quantified utilizing Image Lab Software 6.1 (Bio-Rad). For slot blots, supernatants were collected 72 h after treatment. For each sample, 5 µL supernatant was diluted into 195 µL PBS. These samples were run through a slot blot apparatus (Bio-Rad) with a PVDF membrane and two pieces of filter paper for 5 min, followed by three washes with 1X PBS. The membrane was then blocked with 5% milk in TBST for one hour and probed like a Western blot. All primary antibodies used are listed in Supplemental Table S1 (STS1). Secondary antibodies were obtained from Biorad and were used at a dilution of 1:10,000 for one hour in 5% milk in TBST. For mouse primaries, StarBright520 (12005866) was used and, for rabbit primaries, StarBright700 was used (12004161).

Proliferation Assays: Proliferation was assessed directly and indirectly. Proliferation was directly assessed by trypan blue counts as previously described [28]. As an additional measure, proliferation was also assessed via ATP production. The Cell Titer-Glo kit was purchased from Promega. Primary human lung fibroblasts were plated in 96-well plates and treated with 1 ng/mL TGFβ, 5 ng/mL FGF, or 50 µM BTB for 72 h. At that time, a cell lysis solution containing a proprietary oxyluciferase substrate was introduced. Cells were incubated at room temperature with gentle mixing to ensure complete lysis. Luminescence was then measured with a Tecan Infinite F-Plex plate reader. The luminescent signal produced is proportional to the amount of ATP produced in each well. ATP levels are directly proportional to cell number and thus is often used as an indirect measure of proliferation.

Cytotoxicity Assay: Cytotoxicity was measured by LDH release using the LDH-Glo Cytotoxicity Kit (Promega, J2380, Madison, WI, USA). Supernatants were harvested from cells and stored in an assay compatible storage buffer (200 mM Tris-HCl, 10% glycerol, 1% BSA, pH = 7.3) until the time course was complete. As a positive control, cells were treated with 10% Triton-X for 5–10 min. Complete cell death was confirmed visually to ensure all cells had detached. The samples were incubated with a luciferase reagent mixture for one hour. Upon reduction of luciferase, a luminescent signal was released, which is proportional to the concentration of LDH released. Luminescence was then measured with a Tecan Infinite F-Plex plate reader.

Caspase Activation Assay: The Caspase-Glo assay purchased from Promega contains a pro-luminescent caspase substrate (caspase-3/7 DEVD-aminoluciferin substrate) and a thermostable luciferase. Cells were plated in a 96-well plate format and treated with BTB. Seventy-two hours after treatment, the caspase substrate and reagent were added to induced cell lysis. The cell mixture was incubated for 45 min and intermittently mixed. When caspases are activated, the caspase 3/7 aminoluciferin substrate is cleaved and a luminescent signal is produced. Luminescence was then measured with a Tecan Infinite F-Plex plate reader.

RhoA Activity Assay: RhoA activity was assessed utilizing a Rho Activation Assay Kit from Cytoskeleton (Cat. No. BK036, Denver, CO, USA). This assay exploits the Rho binding domain (RBD) of the effector protein rhotekin. The RBD of rhotekin has a high affinity for the binding domain of GTP-bound RhoA, allowing for affinity-based precipitation. The complete assay was performed at 4 °C to slow the rate of hydrolysis of GTP. Briefly, cells were stimulated with 1 ng/mL of TGFβ to induce activation. Lysates were harvested 15 min after treatment and immediately snap frozen. Ten percent of each lysate was set aside to measure protein concentration. The remaining 90% of lysates were diluted to equal concentrations. Lysates were incubated with RBD-rhotekin coated beads to precipitate active RhoA. Lysates were then run on a Western blot and normalized to total RhoA protein. Positive and negative controls were obtained by incubating lysates with GTPγS (non-hydrolysable GTP) and GDP, respectively. Notably, a high concentration (5 mM) of GDP was required to neutralize basal GTP activity in the negative control.

Statistical Analyses: All data are presented as mean +/− SEM. All experiments were performed in triplicate. Data were analyzed by an unpaired Student’s *t*-test or one-way analysis of variance (ANOVA) where indicated. GraphPad Prism was used for all data analysis. Values of *p* < 0.05 were considered statistically significant.

## 3. Results

### 3.1. Proton Sensing G-Protein Coupled Receptors Are Expressed in Primary Human Lung Fibroblasts

**Figure 1:**
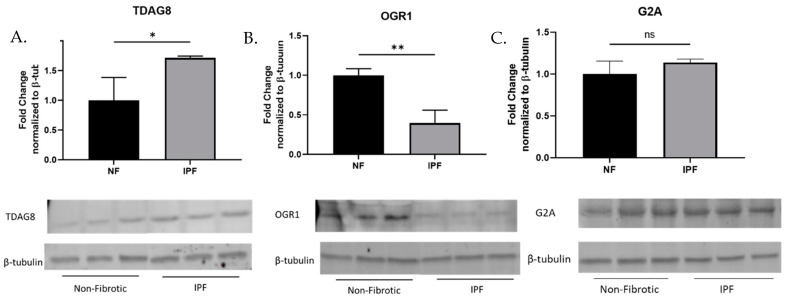
Proton sensing G-protein coupled receptors are expressed in primary human lung fibroblasts. Primary human lung fibroblasts were cultured in six-well plates and grown to a confluency of 85% before protein was harvested to examine expression of TDAG8 (**A**), OGR1 (**B**), and G2A (**C**). Data were analyzed by *t*-test. *n* = 3/group. * *p* < 0.05, ** *p* < 0.01, ns= not statistically significant. All samples were run on the same gel; however, non-contiguous lanes are denoted with a line.

The proton sensing G-protein coupled receptor family includes TDAG8 (GPR65), OGR1 (GPR68), G2A (GPR132), and GPR4. To identify receptors of interest, we examined the protein expression profiles of TDAG8, OGR1, G2A, and GPR4 in primary human lung fibroblasts isolated from patients with or without a diagnosis of Idiopathic Pulmonary Fibrosis (IPF). We hypothesized that receptors that were upregulated in the IPF fibroblasts may be pro-fibrotic and would be prime candidates for drug development. We found that TDAG8 expression was upregulated in IPF fibroblasts (Figure 1A) while OGR1 expression was downregulated in IPF fibroblasts (Figure 1B). G2A expression was not significantly different between healthy and IPF fibroblasts (Figure 1C). GPR4 protein expression was not detected in primary human lung fibroblasts. The varied expression profiles of these receptors in IPF fibroblasts may indicate unique roles for proton sensing receptors in the wound healing process and/or the pathogenesis of IPF.

### 3.2. TDAG8 Knockdown Reduces Markers of Myofibroblast Differentiation

**Figure 2:**
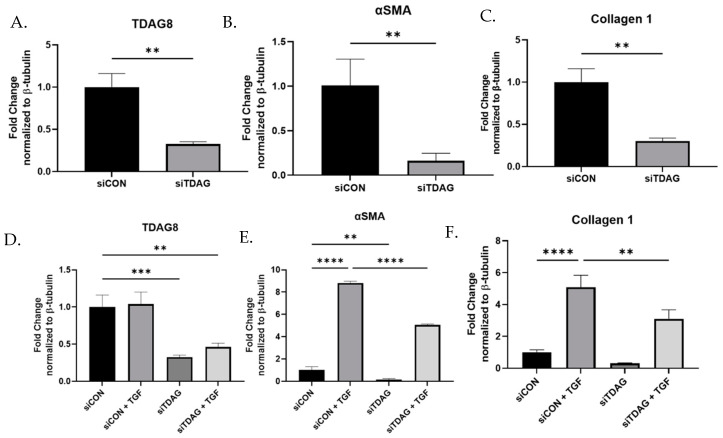

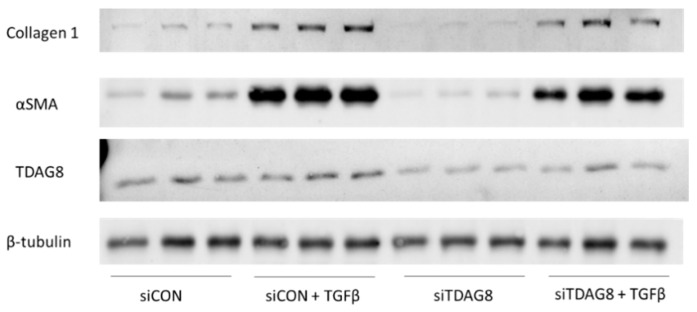
TDAG8 knockdown reduces the ability of TGFβ to induce myofibroblast differentiation. Primary human lung fibroblasts were transfected with non-targeting control siRNA or siRNA specific to TDAG8 for 16 h, upon which media was changed, adding 1 ng/mL TGFβ where indicated, and harvested after 80 h. Lysates were examined for the expression of TDAG8, αSMA and collagen 1 at baseline (**A**–**C**) and in response to TGFβ (**D**–**F**). Data were analyzed by *t*-test, in the case of two groups, or ANOVA where there are multiple groups. *n* = 3/group. ** *p* < 0.01, *** *p* < 0.05 **** *p* < 0.0001.

We hypothesized that proton sensing receptors that were more robustly expressed in IPF fibroblasts may be pro-fibrotic. As TDAG8 was the only receptor exhibiting increased expression in IPF fibroblast, knockdown of TDAG8 was performed to assess if the loss of TDAG8 would be associated with a reduction in pro-fibrotic protein expression (Figure 2A). Knockdown of TDAG8 reduced basal expression of the myofibroblast marker alpha smooth muscle actin (αSMA) (Figure 2B) and the matrix protein collagen 1 (Figure 2C). To identify if the absence of TDAG8 affected myofibroblast differentiation, TDAG8 knockdown was performed in the presence of TGFβ (Figure 2D). TDAG8 knockdown significantly reduced the ability of TGFβ to induce myofibroblast differentiation as measured by the expression of αSMA (Figure 2E) and collagen 1 (Figure 2F).

### 3.3. BTB Prevents Myofibroblast Differentiation

**Figure 3:**
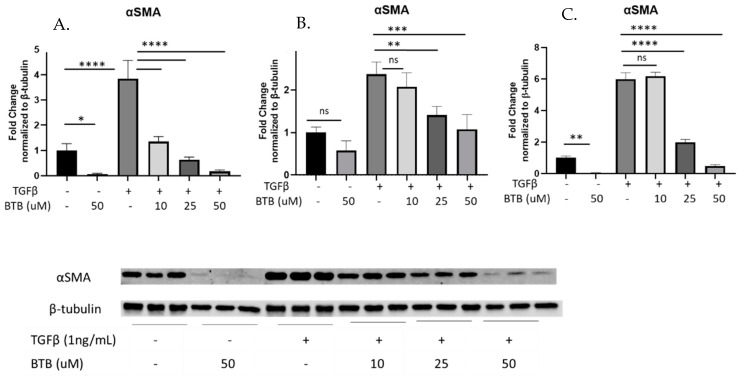

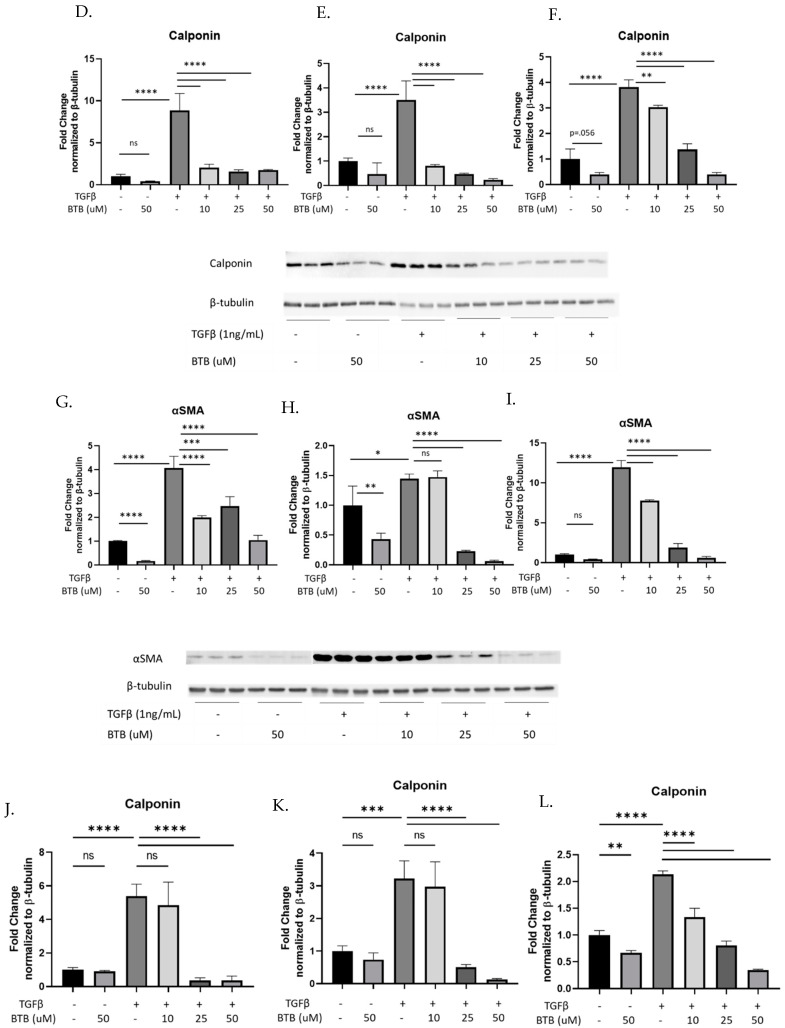

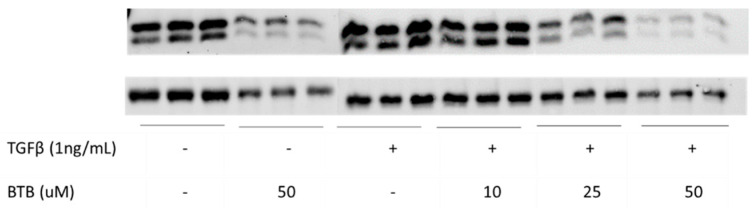
BTB inhibits myofibroblast differentiation in non-fibrotic and IPF-derived fibroblasts. Non-fibrotic and IPF-derived primary human fibroblasts were treated with the indicated dose of BTB and/or 1 ng/mL TGFβ for 72 h before protein was harvested. Each graph represents an individual donor, and all experiments were performed with three non-fibrotic and three IPF-derived cell lines. Non-fibrotic fibroblasts were examined for the expression of αSMA (**A**–**C**) and calponin (**D**–**F**). IPF-derived fibroblasts were also examined for the expression of αSMA (**G**–**I**) and calponin (**J**–**L**). Data were analyzed by ANOVA. *n* = 3 technical replicates/group. * *p* < 0.05, ** *p* < 0.01, *** *p* < 0.001, **** *p* < 0.0001, ns = not statistically significant. Note for gels exceeding 15 samples that the first six lanes of each blot were run on a separately due to the lane capacity of the gel.

There are currently no known inhibitors of TDAG8. However, the novel compound BTB was specifically designed to activate TDAG8 [24]. Given our data demonstrating that TDAG8 knockdown inhibited pro-fibrotic fibroblast phenotypes (Figure 2), we hypothesized that treatment of fibroblasts with BTB would induce myofibroblast differentiation. Contrary to our hypothesis, our data clearly show that BTB inhibits myofibroblast differentiation in primary human lung fibroblasts. We utilized TGFβ (1 ng/mL) in conjunction with a range of doses of BTB (10–50 µM) to assess myofibroblast differentiation over the course of 72 h. BTB effectively inhibited TGFβ induced myofibroblast differentiation as measured by expression of αSMA, in primary human lung fibroblasts isolated from three non-fibrotic donors (Figure 3A–C). Another marker of myofibroblast differentiation, calponin, was also strongly inhibited by BTB (Figure 3D–F). BTB was equally effective at inhibiting myofibroblast differentiation in primary human lung fibroblasts isolated from patients with a diagnosis of IPF. In three IPF fibroblast lines, BTB also inhibited myofibroblast differentiation as assessed by the expression of αSMA (Figure 3G–I) and calponin (Figure 3J–L).

### 3.4. BTB Inhibits Matrix Protein Production in Human Lung Fibroblasts

**Figure 4:**
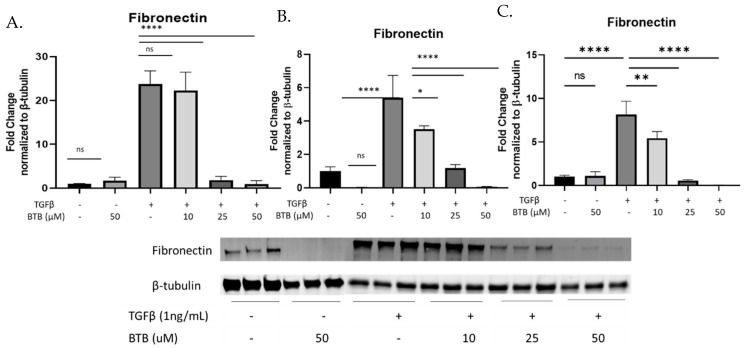

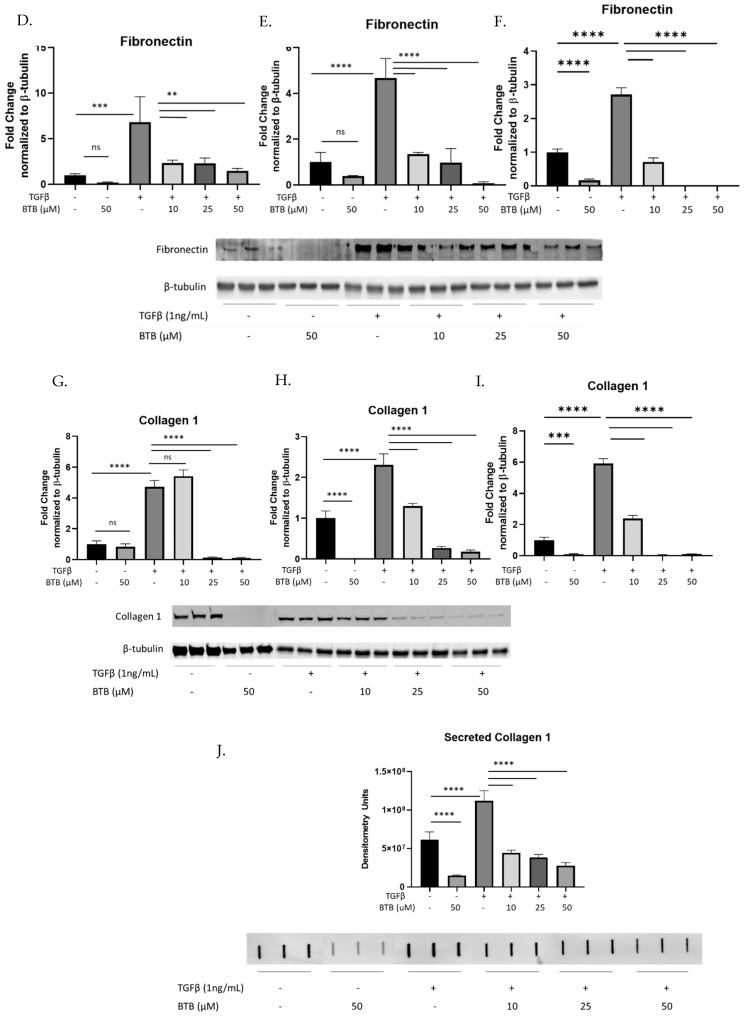

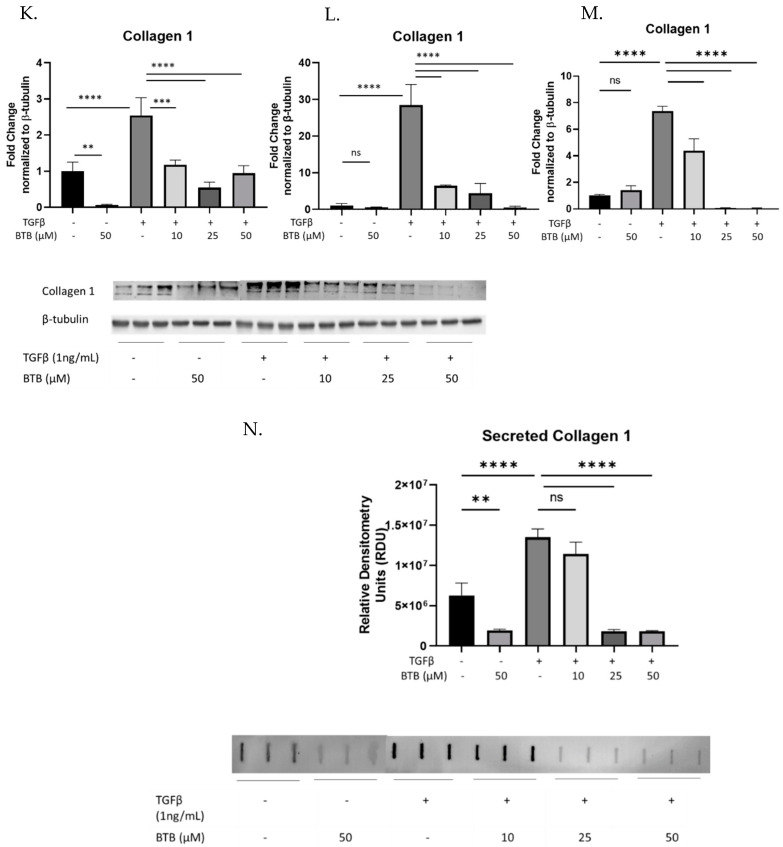
BTB prevents matrix protein production mediated myofibroblast differentiation in non-fibrotic and IPF-derived fibroblasts. Non-fibrotic and IPF-derived primary human fibroblasts were treated with the indicated dose of BTB and/or 1 ng/mL TGFβ for 72 h before protein was harvested. Each graph represents an individual donor, and all experiments were performed with three non-fibrotic and three IPF-derived cell lines. Non-fibrotic and IPF fibroblasts were examined for the expression of fibronectin (**A**–**F**). Collagen protein expression was examined in protein lysates and supernatants from non-fibrotic fibroblasts (**G**–**J**) and IPF derived fibroblasts (**K**–**N**). Data were analyzed by ANOVA. *n* = 3 technical replicates/group. * *p* < 0.05, ** *p* < 0.01, *** *p* < 0.001, **** *p* < 0.0001, ns = not statistically significant. Note for gels exceeding 15 samples that the first six lanes of each blot were run separately due to the lane capacity of the gel.

In addition to the expression of αSMA, myofibroblasts are also distinguished by their ability to produce increased matrix proteins. Fibronectin and collagen 1 are two of the most abundant matrix proteins in the IPF lung, the majority of which is produced by myofibroblasts. BTB inhibits the production of fibronectin (Figure 4A–C) collagen 1 (Figure 4D–F) and secreted collagen 1 (Figure 4G) in non-fibrotic primary human lung fibroblasts. To directly assess the therapeutic potential of BTB, fibroblasts derived from patients with IPF were also utilized. Similar to the non-fibrotic fibroblasts, BTB also inhibits the production of fibronectin (Figure 4H–J), collagen 1 (Figure 4K–M), and secreted collagen 1 (Figure 4N) in IPF-derived fibroblasts.

### 3.5. BTB Inhibits Proliferation

**Figure 5:**
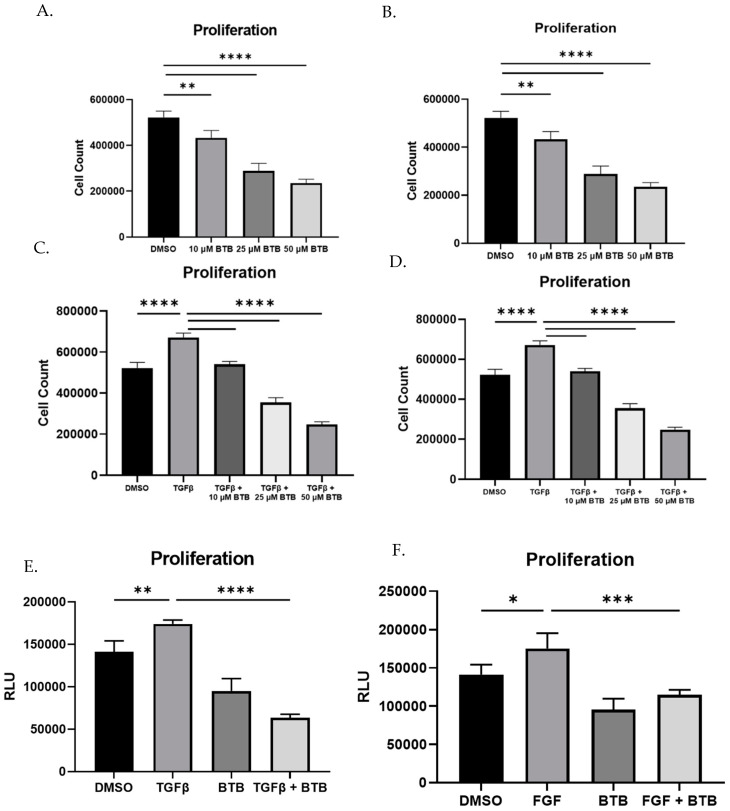

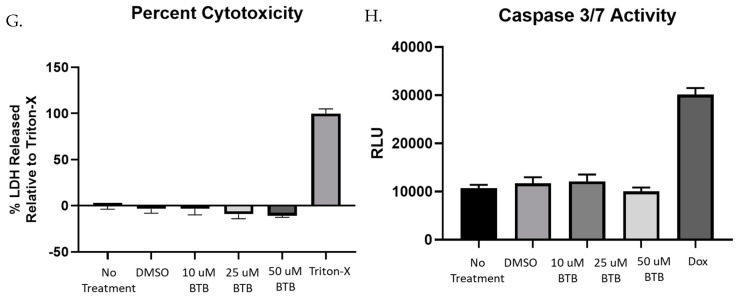
BTB inhibits proliferation without inducing cell death. Fibroblasts were grown in 24 well plates for 72 h with the indicated dose of BTB (50 µM where not indicated), 1 ng/mL TGF or 5 ng/mL FGF. In panels A–D, cell counts were obtained by trypan blue staining and automated cell counting. Cell counts were performed on non-fibrotic and IPF derived fibroblasts at the baseline (**A**,**B**) and in response to TGFβ (**C**,**D**). ATP production was used as an indirect measure of proliferation in non-fibrotic fibroblasts (**E**,**F**). Cytotoxicity was assessed by LDH release (**G**), and a caspase 3/7 activity assay was performed (**H**). Data were analyzed by ANOVA. *n* = 4–6 technical replicates/group. * *p* < 0.05, ** *p* < 0.01, *** *p* < 0.001, **** *p* < 0.0001.

In IPF, there is a marked expansion of the fibroblast population, likely as a result of TGFβ mediated proliferation. BTB inhibits the proliferation of both non-fibrotic and IPF-derived fibroblasts in a dose dependent manner (Figure 5A and Figure 3B). Additionally, BTB is able to inhibit TGFβ induced proliferation in non-fibrotic and IPF derived fibroblasts (Figure 5C and Figure 3D). This result was further confirmed using an indirect measure of proliferation (ATP production). Using the 50 µM dose, BTB was able to inhibit both TGFβ and FGF induced proliferation (Figure 5E,F). To ensure these results were not a result of cytotoxicity or cell death, an LDH release assay was performed over a wide range of doses. There was no cytotoxicity associated with any dose of BTB (Figure 5G), nor was cell death observed over time (Supplemental Figure S1). As an additional method, we also examined if BTB induced apoptosis; however, BTB did not induce caspase 3/7 activity (Figure 5H). Overall, BTB inhibits myofibroblast differentiation, matrix production, and proliferation without the inducing of cell death.

### 3.6. BTB Does Not Require TDAG8 to Inhibit Myofibroblast Differentiation

**Figure 6:**
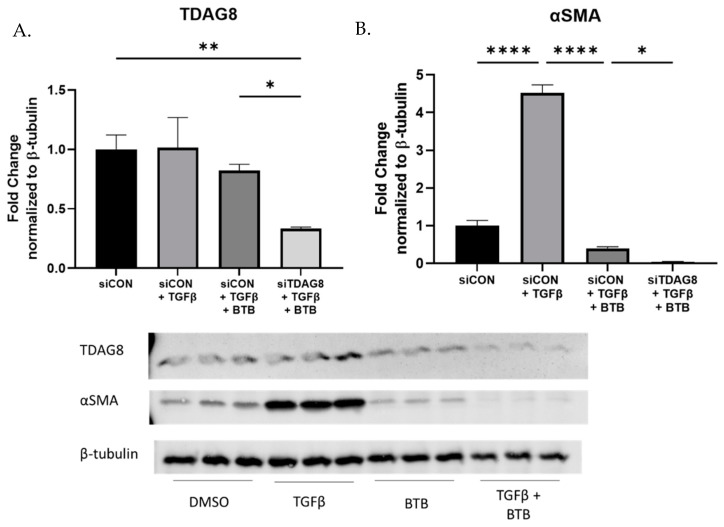
BTB does not require TDAG8 to inhibit myofibroblast differentiation. Fibroblasts were transfected for 18 h, upon which time media was changed. After 54 h, fibroblasts were challenged with 1 ng/mL TGFβ and/or 50 µM BTB. Fibroblasts were incubated for another 48 h prior to protein harvest. TDAG8 knockdown was confirmed (**A**) and lysates were examined for expression of αSMA (**B**). Data were analyzed by ANOVA. *n* = 3 technical replicates/group. * *p* < 0.05, ** *p* < 0.01, **** *p* < 0.0001.

BTB is a novel ligand designed to bind and activate TDAG8. To verify BTB was working via TDAG8 in fibroblasts, TDAG8 was knocked down and then the cells were subsequently challenged with BTB and/or TGFβ. Despite a 70% reduction in TDAG8 expression (Figure 6A), BTB was still effective in inhibiting myofibroblast differentiation (Figure 6B). In fact, when TDAG8 was knocked down, BTB was even more effective. If BTB was functioning exclusively via TDAG8, the reduction in TDAG8 expression should be sufficient to at least blunt the effectiveness of BTB. Our data suggest that the ability of BTB to inhibit myofibroblast differentiation is not related to TDAG8 activation in primary human lung fibroblasts. Both BTB and TDAG8 knockdown inhibit myofibroblast differentiation, but they likely accomplish this via separate mechanisms.

### 3.7. BTB Inhibits the ROCK/Rho Axis

To identify the mechanism(s) by which BTB inhibits the pro-fibrotic behavior of fibroblasts, several signaling pathways were interrogated. Due to the ability of BTB to inhibit the effects of TGFβ, canonical TGFβ signaling was examined. However, BTB did not inhibit Smad2 phosphorylation (Supplemental Figure S2). Following this result, we examined non-canonical TGFβ signaling. 

**Figure 7:**
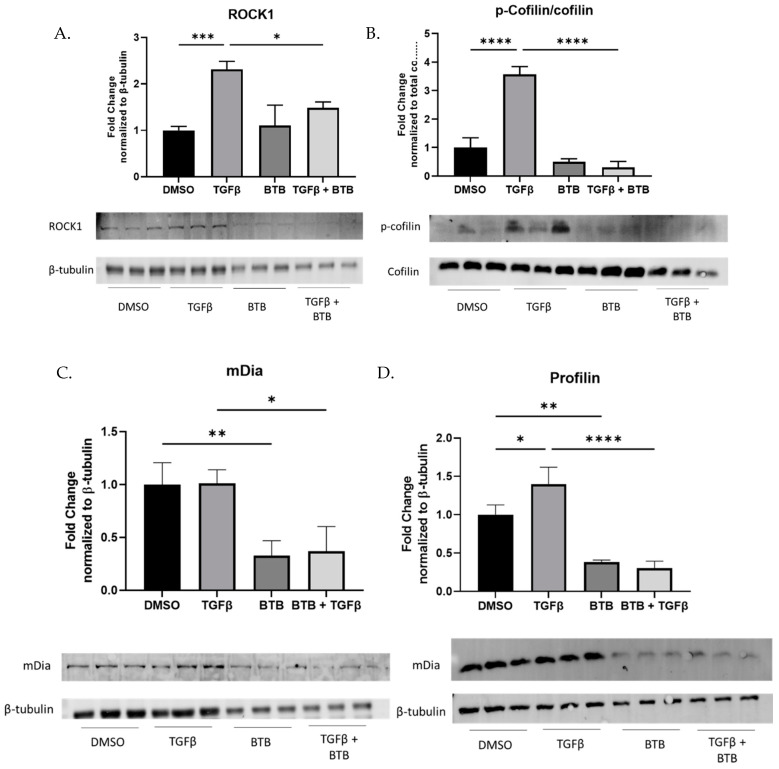

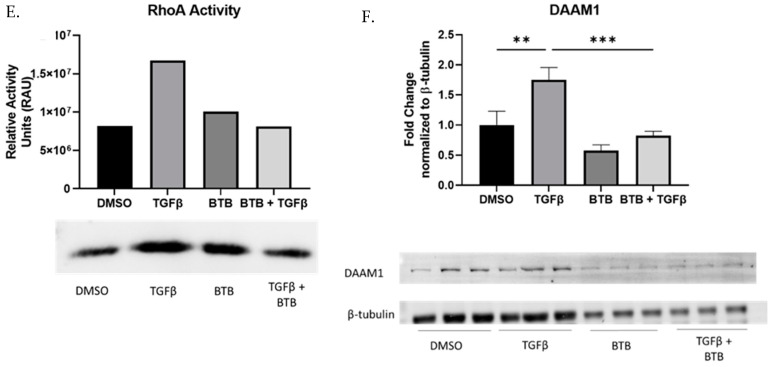
BTB inhibits RhoA mediated signaling. Fibroblasts were treated with 1 ng/mL TGFβ and/or 50 µM BTB for 48 h before protein was harvested. The expression of ROCK1, p-cofilin, mDia, and profilin was examined (**A**–**D**). A RhoA activity assay was performed 20 min after stimulation (**E**) and DAAM1 expression was examined (**F**). Data were analyzed by ANOVA. *n* = 3 technical replicates/group. * *p* < 0.05, ** *p* < 0.01, *** *p* < 0.001, **** *p* < 0.0001.

We next identified the ROCK/Rho signaling axis as a promising mechanism given that this pathway has been identified to induce matrix production [29,30,31], induce proliferation [32,33,34,35], and induce myofibroblast differentiation in vitro [36,37,38]. Importantly, inhibition of ROCK/Rho signaling inhibits fibrosis in vivo [39,40,41,42,43]. ROCK1 is the most well studied effector in this pathway and is known to be pro-fibrotic in human lug fibroblasts. We observe that BTB inhibits ROCK1 protein expression 48 h after treatment (Figure 7A). Cofilin, a downstream mediator of ROCK1, is phosphorylated by ROCK1 and is used as an output of ROCK1 activity [44,45,46]. Forty-eight hours after treatment, BTB also inhibits cofilin phosphorylation (Figure 7B). This indicates that BTB inhibits ROCK1 mediated signaling. However, ROCK1 is not the only effector in this pathway. mDia is the other main effector, which is essential for stress fiber formation. BTB also inhibits mDia and its downstream regulator profilin, at 48 h post-treatment (Figure 7C,D).

**Figure 8:**
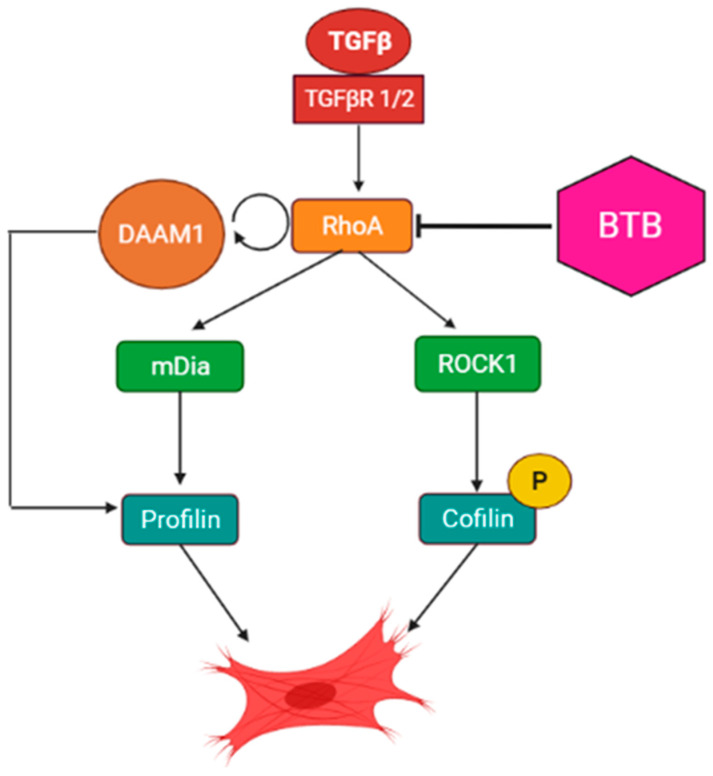
Schematic for the proposed mechanism of the action of BTB. BTB blocks RhoA activation, in part through inhibition of DAAM1. This leads to inhibition of both ROCK1 and mDia signaling, which prevents myofibroblast differentiation.

ROCK1 and mDia are both regulated by the GTPase RhoA [47]. Given the ability of BTB to inhibit both effectors of RhoA, we set out to examine if BTB directly blocks RhoA activity. We performed a RhoA activity assay to determine if BTB could be directly blocking RhoA activity to inhibit this entire signaling axis. After treatment with BTB, we isolated all GTP-bound proteins to precipitate out active RhoA. We found that BTB inhibits RhoA activity, and it is likely that this action is responsible for the ability of BTB to block myofibroblast differentiation (Figure 7E). To further support this hypothesis, we examined the expression of DAAM1, a formin which assists RhoA activation [48,49,50], and found that BTB also inhibits DAAM1 expression (Figure 7F). The published work demonstrates that DAAM1 knockdown is sufficient to inhibit RhoA activity [48,51], which may be one of the mechanisms by which BTB inhibits the ROCK-Rho signaling axis (Figure 8).

## 4. Discussion

IPF is a devastating disease with limited treatment options. Central to the pathogenesis of IPF is myofibroblast differentiation. A number of cellular changes accompany myofibroblast differentiation including increased proliferation, matrix production, and metabolic reprogramming. Our previous studies have centered around how myofibroblasts upregulate glycolytic enzyme expression and produce increased lactate, ultimately inducing myofibroblast differentiation. Lactate is the main byproduct of glycolysis and the primary source of extracellular acidification. As tissue pH is decreased in fibrotic areas of the lung in animal models of pulmonary fibrosis, we began to examine if pH sensing mechanisms could be exploited for therapeutic benefit.

As tissue acidification is associated with fibrosis, we hypothesized that pH-sensing receptors would be upregulated in IPF-derived fibroblasts and that inhibition of these receptors could be anti-fibrotic. We examined the expression profiles of the family of proton sensing GPCRs, identifying TDAG8 as the only proton sensing receptor upregulated in IPF fibroblasts (Figure 1). When TDAG8 was knocked down, markers of myofibroblast differentiation were suppressed at the baseline as well as after treatment with TGFβ (Figure 2).

Given these data, we wanted to assess how pharmacologic manipulation of TDAG8 would affect fibroblast behavior. Although there are no known inhibitors of TDAG8 at this time, a positive allosteric modulator has been identified (BTB09089). As this was the only tool available to manipulate TDAG8 activity, we decided to utilize BTB to illuminate the function of TDAG8 activation in fibroblasts. Given our data, we hypothesized that BTB would activate TDAG8 and thus exacerbate myofibroblast differentiation, but, much to our surprise, we discovered that BTB inhibits myofibroblast differentiation. BTB potently inhibits myofibroblast differentiation, matrix production, and proliferation in primary lung fibroblasts isolated from non-fibrotic and IPF donors (Figure 3, Figure 4 and Figure 5).

Given the unanticipated finding that BTB inhibits myofibroblast differentiation, we set out to confirm whether BTB was indeed inhibiting myofibroblast differentiation through activation of TDAG8. To accomplish this, we knocked down TDAG8 and re-introduced BTB. When TDAG8 was knocked down, BTB was even more efficacious than when TDAG8 expression was normal. This indicates that BTB does not require TDAG8 expression to inhibit myofibroblast differentiation (Figure 6). There are several possible explanations for these data. It is possible that the ability of BTB to bind and/or activate TDAG8 is pH dependent or contingent upon a specific subcellular localization. Additionally, there may be other unknown endogenous ligands that bind to the active site or bind allosterically to modify how BTB interacts with TDAG8. Furthermore, BTB is a small molecule that may be readily taken up by fibroblasts, through passive diffusion or clathrin mediated endocytosis. Neither of these processes would require TDAG8. BTB may also bind to other receptors, potentially within the family of proton sensing receptors, although it is important to note that the research group responsible for BTB did ensure that BTB did not activate the other proton sensing GPCRs. BTB may also activate anti-fibrotic receptors or inhibit profibrotic receptors outside of the family of proton sensing GPCRs. It remains unclear how relevant a TDAG8 dependent mechanism may be in relation to the antifibrotic effect of BTB. Future work will examine if extracellular and intracellular cues, such as altered pH, may drive the interaction between BTB-TDAG8 or if BTB is capable of increasing TDAG8 activity in primary human lung fibroblasts in physiological conditions.

To better understand the mechanism(s) of action of BTB, we examined if BTB inhibited canonical and/or non-canonical TGFβ signaling. Although BTB did not alter Smad2 phosphorylation (Supplemental Figure S2), it did inhibit ROCK/Rho signaling. The ROCK/Rho signaling axis is a known pro-fibrotic pathway and has been identified as a mechanosensitive pathway which controls actin dynamics and myofibroblast differentiation. However, there are several elements of this pathway that remain understudied. BTB effectively inhibits both ROCK1 and RhoA, as well as their downstream mediators, cofilin, and profilin, respectively (Figure 7). This led us to hypothesize that BTB may be inhibiting RhoA directly. Indeed, we found that BTB inhibits RhoA activity. We propose that this is how BTB inhibits mDia and ROCK1 signaling, ultimately preventing myofibroblast differentiation. In further support of this hypothesis, we also found that BTB inhibits DAAM1, which is a formin that supports RhoA activation (Figure 7). Other researchers have found that knockdown of DAAM1 is sufficient to inhibit RhoA activity. These two findings strongly suggest that BTB directly targets the RhoA signaling axis to inhibit differentiation. An integrated schematic for the proposed mechanism of action of BTB is shown in Figure 8.

This manuscript, in conjunction with other work, highlights several interesting future directions. TDAG8 is a prime candidate for study, both in basic science and clinical research. First, the novel finding that TDAG8 knockdown is anti-fibrotic in fibroblasts warrants further investigation. Published work on TDAG8 has largely focused on inflammation in the epithelium, which suggests that TDAG8 activation is anti-inflammatory while knockdown of TDAG8 is pro-inflammatory [52,53,54]. This is of particular interest in fibrosis, where the balance between inflammation and fibrosis has been a topic of ongoing discussion. The potential for targeted therapies which regulate the balance of inflammatory and fibrogenic signaling in epithelial cells versus fibroblasts would be a major achievement in fibrosis research.

In regard to clinical research, TDAG8 is a genetically diverse receptor with several single nucleotide polymorphisms (SNPs). In the context of IBD, specific TDAG8 genotypes correlated with disease severity [55]. Examination of candidate genes, such as TDAG8, in the context of IPF pathobiology is now possible given the launch of the first precision medicine clinical trial. Genetic contributions to the incidence and progression of IPF are well established. A more complete understanding of individual genetic variation may significantly affect disease progression and medication response.

In recent years, publicly available databases containing wide-scale sequencing information in IPF cohorts have greatly expanded our ability to interrogate associations between pathobiology and genetics in a cell-type specific fashion [56,57,58,59]. Foundational genetic studies lead the way for detailed functional analysis of resulting proteins across cell types in health and disease. In these databases, TDAG8 expression is low in fibroblasts and myofibroblasts compared to other immune cell populations. However, it is important to consider that transcriptional changes do not always result in altered translation. Thus, genetic and proteomic studies should be conducted in parallel to understand the relative contributions of transcriptional versus translational regulation.

BTB represents a prime candidate for future therapeutic development. This paper identifies how BTB interferes with RhoA mediated signaling; however, more work is needed to determine its precise mechanism of action. Additionally, other pro-fibrotic pathways, such as Wnt, converge with RhoA signaling [60] and may work in conjunction to drive fibrotic changes in the lung. BTB may serve to inhibit multiple nodes of pro-fibrotic signaling pathways to accomplish such striking results.

From this study, we conclude that BTB has strong therapeutic potential as it potently inhibits myofibroblast differentiation. Future work will examine if BTB is an effective treatment for pulmonary fibrosis in vivo by utilizing a TGFβ-adenoviral model and a bleomycin induced model of fibrosis. This work also indicates that TDAG8 knockdown has anti-fibrotic potential. Future studies will focus on how TDAG8 may regulate the balance between inflammation and wound healing as it relates to pathogenesis of fibrosis.

## Data Availability

Not applicable.

## Supplementary Materials

The following supporting information can be downloaded at: https://www.mdpi.com/article/10.3390/ijms231911946/s1.
