# Supplementary material for: The Novel Small Molecule BTB Inhibits Pro-Fibrotic Fibroblast Behavior though Inhibition of RhoA Activity
Source: Int J Mol Sci. 2022 Oct 8;23(19):11946. doi: 10.3390/ijms231911946 (PMC9569993; doi:10.3390/ijms231911946)
Supplement: Supplementary file 1 [file ijms-23-11946-s001.zip › ijms-1910973-supplementary.pdf]

BTB Paper Supplemental Figures

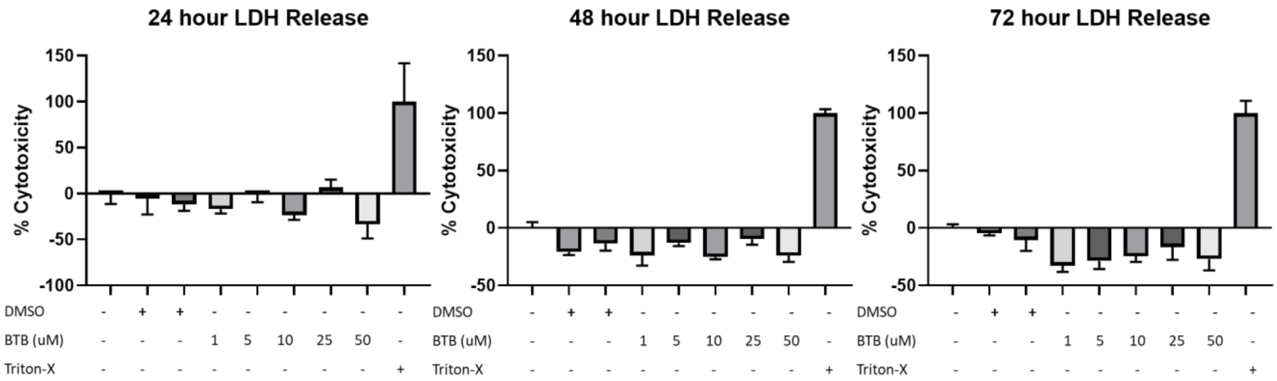

Supplemental Figure S1 (SF1). BTB Does Not Induce Cell Death Over 72 hours. Fibroblasts were cultured on 96 well plates for 72 hours. Media was removed every 24 hours for 72 hours (the cumulative removed media volume never exceeded 10% of the total well volume) and assayed for LDH content.

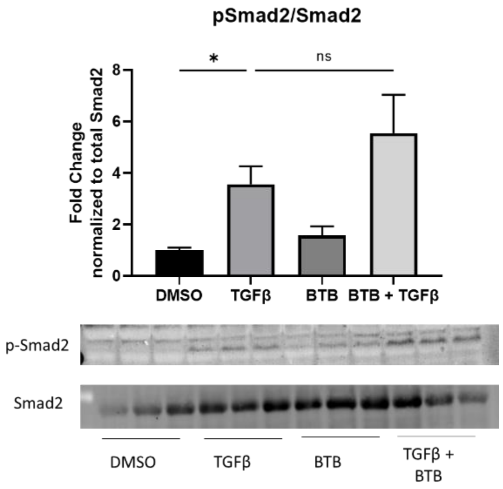

Supplemental Figure S2 (SF2). BTB Does Not Inhibit Smad2 Phosphorylation. Fibroblasts were treated with 1 ng/mL TGFβ and/or 50 μM BTB for 60 minutes before protein was harvested to examine Smad2 phosphorylation. Data was analyzed by ANOVA, n=3/group, *=P<.05.

Supplemental Table S1. Primary Antibodies.

Antibody	Company	Catalogue Number
AIF	Cell Signaling Technology	4642
Alpha smooth muscle actin-Cy3	Sigma	C6198
Beta-tubulin-Cy3	Abcam	Ab11309

Calponin	Abcam	Ab46794
Cofilin	Cell Signaling Technology	5175S
Cofilin	Santa Cruz	SC-376476
Collagen 1	Aviva	ARP59999
DAAM1	Santa Cruz	SC-100942
Fibronectin	Abcam	Ab6328
Gβ	Santa Cruz	SC-166123
Histone H3	Cell Signaling Technology	9715
HSP90	Cell Signaling Technology	4874
Lamin A/C	Cell Signaling Technology	4777
mDia	Thermo Fisher Scientific	20624-1-AP
p-cofilin	Cell Signaling Technology	3313S
Profilin	Santa Cruz	SC-137235
pSmad2	Abcam	188334
RhoA	Cell Signaling Technology	2117S
ROCK1	Abcam	EPR638Y
Smad2	Cell Signaling Technology	3103S
TDAG8	Sigma	SAB29000463
